# Navigating the Precipice: continuous renal replacement therapy in critically Ill children with oncologic diseases

**DOI:** 10.1016/j.jped.2026.101505

**Published:** 2026-01-30

**Authors:** Shina Menon

**Affiliations:** Stanford University, Department of Pediatrics, Division of Nephrology, Palo Alto, United States of America

Multiple organ dysfunction syndrome (MODS), characterized by a dysregulated systemic inflammatory process, is common in the pediatric intensive care unit (ICU), occurring in nearly 20% of children on admission, and is associated with a higher risk of death and worse functional outcomes [1,2]. In pediatric cancer patients, MODS may be secondary to a complex interplay of factors related to both the underlying malignancy and its treatment, with sepsis/septic shock being the leading cause. A subset of patients with MODS and severe acute kidney injury (AKI) require renal replacement therapy (RRT), and the outcomes are worse in these patients [3,4].

In the article, "The role of continuous renal replacement therapy in critically ill children with cancer and multiple organ dysfunction syndrome", Veiga et al. present a retrospective cohort study, conducted over a six-year period (2013-2019) in a dedicated pediatric oncology ICU in São Paulo, Brazil [5]. They describe the characteristics and outcomes of 59 children with cancer and MODS who required CRRT. The cohort included 18 (30%) patients with a history of hematopoietic stem cell transplantation (HSCT), a median (interquartile range, IQR) of 3 organ systems involved, and almost 80% were receiving mechanical ventilation. Nearly half (44%) had a positive fluid balance (FB) > 10% in the 72 hours prior to CRRT and the median duration of CRRT was 9 (4.5-15) days. Hospital mortality was only 67.7%.

This study highlights how patients with a history of malignancy of HSCT differ from other critically ill children receiving CRRT in the ICU. In a multicenter cohort study from Worldwide Exploration of Renal Replacement Outcomes Collaborative in Kidney Diseases (WE-ROCK) that included nearly 1000 children and young adults receiving continuous RRT (CRRT), the median (IQR) CRRT duration was 6 (3, 14) days, and ICU mortality was 36% [6]. In a secondary analysis from the same cohort that evaluated 446 patients with sepsis at CRRT initiation, those with sepsis had longer duration of CRRT (7 days vs 5 days, p 0.026) and higher incidence of in-hospital mortality (47% vs. 31%, p < 0.001) compared to those without sepsis [7]. While the mortality is worse compared to other ICU cohorts, it aligns with data from other studies report mortality rates between 50% and 70% for similar cohorts of pediatric oncology or hematopoietic stem cell transplant patients requiring CRRT [3,4]. In a multicenter study from 8 pediatric ICUs in the Netherlands, Raymakers-Janssen et al. reported on 1,927 admissions with pediatric cancer or HSCT including 68 who received CRRT. The mortality of patients requiring CRRT was 54.4% compared to 11.2% overall [3]. FB (odds ratio, 1.08; 95% CI, 1.01-1.17) and need for inotropic support (odds ratio, 6.53; 95% CI, 1.86-23.08) at the start of CRRT were associated with PICU mortality. Veiga et al. show that mechanical ventilation (OR: 8.48) and FB in the final 72 hour prior to CRRT (OR: 1.15) were associated with mortality [5].

Using laboratory data, Veiga et al. demonstrated that CRRT was technically effective, with significant improvements in biochemical markers within the first 72 hours of therapy [5]. They also showed a significant improvement in the FB during CRRT among both survivors and non-survivors. This confirms what we know CRRT can do- slowly and meticulously correct the fluid and electrolyte balance in severe AKI. However, the fundamental question that remains is, whether this restored homeostasis translates into better outcomes? This is particularly relevant with respect to FB and pathologic fluid accumulation. While FB at CRRT initiation is often associated with mortality, fluid removal during CRRT may have limited impact [[8], [9], [10]]. In a single center observational study of pediatric patients receiving extracorporeal membrane oxygenation (ECMO) and CRRT, after adjusting for FB at CRRT initiation, age, and severity of illness, the change in fluid balance at CRRT discontinuation was not significantly associated with mortality (p = 0.212) [10]. Models investigating rates of fluid removal found that FB at CRRT initiation was the most consistent predictor of survival, and correction to ≤ 10% was not associated with improved survival [10]. Similar results have been seen in other cohorts. Thus, suggesting that preventing the development of pathologic fluid accumulation and interventions prior to the development of significant positive FB may be more clinically effective than attempting fluid removal after [10,11].

This raises a crucial, and often debated, question: the timing of initiation. While this study was not designed to answer this question, it provides context for the debate. Should we initiate CRRT earlier, proactively, to *prevent* severe fluid overload, rather than waiting to *treat* its consequences? The optimal timing for initiating RRT in both adult and pediatric patients remains a subject of debate. Large, randomized studies in adults, typically using onset of severe AKI or biochemical criteria to define timing, have failed to demonstrate a consistent survival benefit to “early” initiation [12,13]. In children, data are limited to retrospective observational studies. In a single center study, Modem et al. showed that the time to CRRT initiation was significantly lower in survivors compared to non-survivors (2 vs. 3.4 days) [14]. Cortina et al. showed that the odds of mortality increased by 1% for every hour of delay in CRRT initiation [15]. In a multicenter retrospective study from WE‑ROCK which included 969 patients from birth to 25 years of age, each one-day delay in CRRT initiation after ICU admission was associated with a 3% increase in the odds of major adverse kidney events at 90 days (MAKE-90) and a 4% increase in mortality [16]. The optimal thresholds of when the patient needs CRRT, what degree of fluid balance is harmful can be sometimes hard to define, and one need to balance the risks associated with extracorporeal therapy, especially vascular access in patients who are at much greater risk of infection and bleeding, with the benefits.

Another question that comes up is if there are other extracorporeal therapies that may help improve the outcomes of these children in addition to CRRT [17]. While newer filters are starting to be available, most are not approved in the pediatric age group, and the available pediatric data remains limited to case series and small observational studies, with no randomized controlled trials in children [[18], [19], [20]]. However, despite improvement in illness severity scores and biochemical parameters, mortality remains unchanged [19]. More recently the selective cytopheretic device (SCD, SeaStar Medical, Denver, CO, USA) has received approval under humanitarian device exemption from the United States Food and Drug Administration for use in pediatric patients (weight ≥ 10 kg and age ≤ 22 years) with AKI due to sepsis or a septic condition on antibiotic therapy and requiring RRT. The device immunomodulates activated circulating leukocytes and provides a new therapeutic approach to systemic inflammatory response syndrome and AKI and has shown promising results in 2 small multicenter cohorts [21]. A recent European consensus statement emphasizes that current evidence consists primarily of case reports and small case series, with lack of high-quality data being the main limitation [22]. Future research should focus on patient stratification to identify those most likely to benefit and on conducting large multicenter studies.

Moving forward we need to think beyond hospitalization survival and focus on long-term outcomes. In the study be Veiga et al. only 1 survivor with history of Wilms tumor required dialysis after discharge, but other studies have described high incidence of long term chronic kidney disease in this population [5,23]. A study looking at functional status scales in over 500 children and young adults on CRRT from WE-ROCK showed that 18% patients acquired a new morbidity at discharge [24]. Understanding the long-term morbidity in survivors is essential for truly appreciating the net benefit of our interventions and for counseling families appropriately.

It is also imperative to recognize the context in which this study was performed. The authors report from a specialized center in Brazil, highlighting the fact that these complex clinical challenges are universal. Providing CRRT is a resource-intensive endeavor, requiring specialized equipment and consumables, highly trained nursing staff, and collaboration between nephrologists and intensivists. The ability to successfully implement and sustain such a program, as demonstrated by Veiga and colleagues, is a monumental achievement and offers valuable insights for other centers in similar settings.

The study by Veiga and colleagues is a vital report from the front lines of pediatric critical care onconephrology [5]. It serves as a validation of CRRT as an essential tool for managing the life-threatening metabolic and fluid disturbances in children with cancer and MODS. Simultaneously, it is a sobering reminder that our most advanced supportive technologies are often insufficient in the face of overwhelming disease. The high mortality reported is a measure of the profound challenge this patient population represents. This work should not lead to therapeutic nihilism, but rather to a renewed sense of purpose. It calls on us to improve how we identify patients at high risk of developing severe, progressive AKI and initiating therapy in a timely manner [25]. It exhorts us to be better at conducting the rigorous, prospective, multicenter research needed to refine our techniques and answer fundamental questions about timing, dose, and modality. It underscores the importance of fostering global collaborative networks to share data, protocols, and expertise to improve outcomes for all children, regardless of geography [26].

## Conflicts of interest

The author declares no real or perceived conflicts of interest pertinent to this editorial. For full disclosure, here is an additional list of commitments and funding sources that are not directly related to this study: Shina Menon is a consultant for Medtronic, Inc and Nuwellis, Inc, and has research funding from the Gerber Foundation. No other competing interests were reported.
